# Understanding Idiopathic Interstitial Pneumonia: A Gene-Based Review of Stressed Lungs

**DOI:** 10.1155/2015/304186

**Published:** 2015-10-11

**Authors:** Coline H. M. van Moorsel, Thijs W. Hoffman, Aernoud A. van Batenburg, Dymph Klay, Joanne J. van der Vis, Jan C. Grutters

**Affiliations:** ^1^Center for Interstitial Lung Disease, Department of Pulmonology, St. Antonius Hospital, P.O. Box 2500, 3430 EM Nieuwegein, Netherlands; ^2^Division of Heart and Lung, University Medical Center Utrecht, P.O. Box 85500, 3508 GA Utrecht, Netherlands; ^3^Center for Interstitial Lung Disease, Department of Clinical Chemistry, St. Antonius Hospital, P.O. Box 2500, 3430 EM Nieuwegein, Netherlands

## Abstract

Pulmonary fibrosis is the main cause of severe morbidity and mortality in idiopathic interstitial pneumonias (IIP). In the past years, there has been major progress in the discovery of genetic factors that contribute to disease. Genes with highly penetrant mutations or strongly predisposing common risk alleles have been identified in familial and sporadic IIP. This review summarizes genes harbouring causative rare mutations and replicated common predisposing alleles. To date, rare mutations in nine different genes and five risk alleles fulfil this criterion. Mutated genes represent three genes involved in surfactant homeostasis and six genes involved in telomere maintenance. We summarize gene function, gene expressing cells, and pathological consequences of genetic alterations associated with disease. Consequences of the genetic alteration include dysfunctional surfactant processing, ER stress, immune dysregulation, and maintenance of telomere length. Biological evidence shows that these processes point towards a central role for alveolar epithelial type II cell dysfunction. However, tabulation also shows that function and consequence of most common risk alleles are not known. Most importantly, the predisposition of the *MUC5B* risk allele to disease is not understood. We propose a mechanism whereby MUC5B decreases surface tension lowering capacity of alveolar surfactant at areas with maximal mechanical stress.

## 1. Idiopathic Interstitial Pneumonia

Idiopathic interstitial pneumonias (IIP) are a class of diffuse lung diseases comprising several distinct entities. Idiopathic pulmonary fibrosis (IPF) is the most common and severe form of IIP. Median survival in IPF is 3 years [1]. Other less common entities include nonspecific interstitial pneumonia (NSIP), desquamative interstitial pneumonitis (DIP), and cryptogenic organizing pneumonia (COP). Distinction between the different entities of IIP is important with regard to prognosis and therapeutic decision-making, including timing of lung transplantation or palliative care. However, genetic discoveries have raised the question whether the various types of IIP are in fact different disease manifestations within the same pathogenetic spectrum [2]. In a large cohort of patients with familial interstitial pneumonia (FIP), it was found that a diagnosis of IPF was most frequent, but all subtypes of IIP were represented [3]. Furthermore, although it is commonly assumed that IPF* does not* and non-IPF IIP* does* respond to immunosuppressive treatment, part of the non-IPF IIP patient population are refractory to treatment and progress to end-stage fibrosis with severely reduced survival [4].

## 2. Familial Disease

All human phenotypes, including disease phenotypes, are influenced by a person's genetic constitution. In case of IIP, evidence for a more defining genetic contribution to disease is most compelling. Ethnic differences in incidence of IPF include higher occurrence in Hispanics than in Whites and the lowest occurrence in Blacks and Maori [5, 6]. In theory, familial occurrence may well be explained by presence of a common environmental cause. An environmental cause requires clustering of affected family members in space and time, while a genetic cause allows for differences in space and time. Such differences are frequently observed in familial IIP including sibs from different environments and parent-offspring disease with an interval of decades [3, 7–9] and support the involvement of heritable factors. IIP is familial in approximately 10% of cases [10] and might even reach 20% in cohorts with IPF or end-stage lung disease [11, 12]. These numbers may even be an underestimation, because the studies relied on patient reports. With more elaborate measurement of familial disease, an even larger familial component can be identified. Scholand and coworkers performed an extraordinary study for which they first identified 1,000 cases that died from pulmonary fibrosis in the Utah Population Database. They showed that the average relatedness of these 1,000 cases was significantly higher than that of matched controls* even* when first- and second-degree relatives were excluded [13].

## 3. Alveolar Epithelial Type II Cell

A major breakthrough was achieved when the first causative mutation was identified in a family with IIP. Candidate gene sequencing detected a heterozygous mutation in surfactant protein C (*SFTPC*) [14]. Because* SFTPC* is exclusively expressed in type II alveolar epithelial cells (AECs), it was proof that erroneous processes in AEC type II could ultimately lead to pulmonary fibrosis.

The reported family already contained many features of disease associated with* SFTPC* mutations: familial ILD, dominant expression, variable penetrance, and expressivity resulting in acute and chronic lung disease in individuals ranging from newborn to adult [10, 15, 16]. Since the first discovery, many IIP families with surfactant mutations have now been described. SFTPC mutations are now established as an important cause of paediatric ILD but also known to contribute to, predominantly familial, IIP in adults [10, 17–19].  Table 1 summarizes characteristics of mutated genes in IIP and biological consequences of mutations.

## 4. Surfactant Processing 

After transcription and translation of SFTPC in AEC type II, a proprotein is formed which after subsequent folding and cleavage steps becomes a mature surfactant protein ready for secretion into the alveolar space via lamellar bodies. Pulmonary surfactant consists of a mixture of lipids and specific proteins that lowers alveolar surface tension thereby preventing alveolar collapse at the end of expiration [20, 21].

Erroneous SFTPC processing is currently one of the best studied mechanisms leading to IIP. The consequence of a mutation in* SFTPC* depends on its position in the gene [22].

Mutations in the C-terminal BRICHOS domain generally increase endoplasmic reticulum (ER) stress and activate the unfolded protein response (UPR) in AEC type II [23–26]. In turn, ER stress can induce epithelial-to-mesenchymal transition in lung epithelial cells [24].

The role of ER stress is not limited to SFTPC mutation carriers, as different studies showed that AECs in fibrotic tissue from nonmutated FIP and sporadic IPF patients were also positive for ER-stress markers [23, 27].

The most common* SFTPC* mutation is I73T and does not cause substantial elevation of ER stress [28]. It represents a linker domain mutation that alters trafficking of the propeptide to early endosomes [29] and causes dysregulated proteostasis [30]. Furthermore, alteration of the surfactant lipid composition and activation of immune cells are reported for these mutations [31].

Later, mutations in a second surfactant associated gene, surfactant protein A2 (SFTPA2), were identified in two families. Family members presented with adult early-onset pulmonary fibrosis or lung cancer with features of bronchioloalveolar carcinoma [32]. Surfactant protein A2 is a C-type lectin important in the defense against respiratory pathogens and in the lung. It is expressed not only by AEC type II, but also by Clara cells and submucosal glands [33, 34]. The mutant protein, when expressed in AECs, was not excreted but retained in the ER and induced ER stress [35], a process similar to that seen in SFTPC mutants. However, there are no reports on lung cancer in patients with SFTPC mutation. ER stress is linked to tumorigenesis [36] and tumorigenesis in BAC is often thought to involve Clara cells [37].

## 5. Lamellar Bodies

Lamellar bodies are secretory organelles unique to type II AEC and are crucial for biosynthetic processing and transport of pulmonary surfactant. Proteins of the limiting membrane of lamellar bodies are encoded by the gene* ABCA3*. In the lung, the highest expression of* ABCA3* has been observed in type II AEC and corresponds with the presence of lamellar bodies [20]. Recessive mutations in* ABCA3* are the most common genetic cause of lethal surfactant deficiency in neonates or chronic ILD in children [38–40].

In type II AECs,* ABCA3* mutations cause abnormal processing, trafficking, and functionality of the ABCA3 protein [41, 42], resulting in impaired lipid transport [43] or retention in the ER compartment and elevated ER stress and apoptotic signaling [44]. Only recently were compound heterozygous or homozygous mutations in* ABCA3* described in adult IIP [45, 46]. A French patient with* ABCA3* mutations presented with combined pulmonary fibrosis and emphysema (CPFE) [46]. CPFE typically occurs in male smokers but also has similarities to radiographs of IIP patients with* SFTPC* mutations [47]. Dysfunctional lamellar bodies in AEC type II were also identified as a cause of pulmonary fibrosis in Hermansky-Pudlak Syndrome (HPS).

HPS is a systemic disorder characterized by reduced pigmentation of skin, hair, and eyes and bleeding diathesis. Disease is caused by autosomal recessive mutations in the gene* HPS1 *[48] that lead to giant lamellar bodies in type II AECs with a deficiency to fuse with the outer cell membrane and excrete lamellar body content [49–51]. Pulmonary fibrosis in HPS shares many similarities with that observed in IPF [52, 53]. Due to unfamiliarity with the disease and the variable degree of albinism and bleeding disorders misdiagnosis might occur. However, clinical features characteristic for HPS are scars in pulmonary fibrosis. In a cohort study including 127 patients with pulmonary fibrosis, only four patients had two or more features consistent with HPS. One out of four had HPS and was compound heterozygous for mutations in* HPS1. *Review of her medical documentation showed that she had received a diagnosis of IPF prior to referral to a tertiary center [54].

## 6. Telomere Maintenance

A different set of genes involved in FIP was discovered when anamnestic familial [55] and genome-wide linkage [56] analysis linked IIP to mutations in the genes TERT and TERC. The gene* TERT* encodes telomerase reverse transcriptase, which together with the transcript of the telomerase RNA component (*TERC*) forms the telomere complex, required to maintain telomere length.

Mutations in the telomerase genes have been found to cause a telomere syndrome with one or more manifestations of early aging, such as idiopathic pulmonary fibrosis, bone marrow failure, or cryptogenic liver cirrhosis [57]. A mutation in one* TERT* allele can lead to haploinsufficiency that results in overall decreased telomerase activity and is manifested as premature aging disorders. Approximately half of the mutations (10 out of 19) have less than 40% loss of telomerase function. In a heterozygous individual carrying one wild type and one mutant allele, this would result in “normal,” >80%, overall telomerase activity [58]. However, carriers of mutations that cause a minor decrease in overall telomerase activity were shown to cause significant reduction of telomere length over generations that will eventually lead to telomere syndromes [55, 56, 59]. It is not the presence of the mutation* per se* but the length of telomeres that confers the risk for disease. Because telomere length is heritable, carriage of slightly dysfunctional alleles will over generations lead to pathologically short telomeres, a phenomenon known as genetic anticipation.

Leukocytes telomere lengths in cohorts of FIP and sporadic IIP patients were significantly shorter compared to age-matched controls [60, 61]. Although this is interesting, acquired telomere shortening is a common feature of disease in humans and, recently, it was shown that all ILD patient cohorts have shorter telomere length than controls. However, patients with sporadic IPF had significantly shorter telomeres than patients with other forms of IIP or patients with surfactant mutations [62]. This suggests that telomere shortening is a common denominator of patients with ILD but telomere dysfunction is only key to IPF.

## 7. Alveolar Epithelial Type II Cell Senescence

Although telomerase has much more functions than maintenance of telomere length, these are not suggested to play a major role in the pathogenesis of IIP. The prominent role of telomere length instead of telomerase is underlined by the observed genetic anticipation in congruence with development of disease phenotypes. Furthermore, recently, another four genes,* TINF2*,* DKC1*,* RTEL1*, and* PARN*, involved in telomere maintenance have been discovered to harbor mutations associated with IIP [63–67]. Altogether, this points towards maintenance of telomere length as the unifying cause and not the secondary functions of the individual genes.

AECs type II are responsible for growth, differentiation, and repair in alveoli [68]. In case of alveolar injury AEC type II cells proliferate along the alveolar basement membrane and differentiate in AEC type I [69].

Recently, it was shown that critically short telomeres in AEC type II preferentially induce cellular senescence [70]. Such cell cycle alterations are mediated by p53 and p21. Increased levels of p53 and p21 have been observed in hyperplastic AECs in IPF patients [71] and polymorphisms in these genes were shown to associate with disease development in IPF [72]. Cellular senescence of type II AEC caused regenerative defects, inflammatory responses, and susceptibility to injury in mice lungs and mice lung organoids [70]. This strongly implicates type II AEC senescence as a causative mechanism in IIP pathogenesis.

## 8. Common Risk Alleles

At the outset of genetic studies at the end of the last century, pulmonary fibrosis was thought to result from a chronic inflammatory process. It was therefore logical that cytokine genes were among the first candidate genes studied [73]. Only one gene from that period, interleukin-1 receptor antagonist (*IL1RN*), the gene encoding interleukin-1 receptor antagonist (IL-1Ra), now fulfills our criterion of independent replication, although both positive and negative associations have been published [74–77]. A meta-analysis including all five cohorts showed that carriage of* IL1RN *VNTR^*∗*^2 predisposed to IPF with an odds ratio of 1.6 [78]. The risk allele associates with a reduction of the IL-1Ra to IL-1*β* ratio and thereby causes a profibrotic environment.* In vivo* this effect can be counteracted with addition of IL-1Ra, which was shown to prevent fibrogenesis in mice with bleomycin induced fibrosis [79]. Mutations in* IL1RN* cause deficiency in IL-1Ra which result in systemic life-threatening neonatal autoinflammatory disease [80]. Local deviations of desired IL-1Ra levels might be associated with the autoinflammatory environment that is seen in IPF lung and is not responsive to immunosuppressive therapy. Immunosuppressive therapy suppresses IL-1Ra synthesis [81] and has been shown to be harmful in IPF [82]. All other evidence for the involvement of common genetic variants in IIP is the result of hypothesis-free genome-wide studies. The common genetic variants that have been associated with IIP in multiple independent cohorts are shown in Table 2.

## 9. Genome-Wide Studies

Genome-wide linkage and fine-mapping identified the minor allele of rs35705950 to be associated with disease in both FIP and IPF. rs35705950 is situated in the putative promoter of* MUC5B* and the risk allele was shown to correlate with increased* MUC5B* expression in lung from unaffected subjects [83]. Carriage of the risk allele conferred high odds ratios well over five [83, 84]. Such high odds ratios in genome-wide analyses are seldom found and would usually involve rare variants [85]. However, the minor allele of rs35705950 is not rare in Caucasian cohorts where the population frequency is approximately 10%.

In African Yoruban, African American, and Asian populations, risk allele frequencies are rare and vary between 0 and 3% (http://www.ncbi.nlm.nih.gov/projects/SNP/snp_ref.cgi?rs=35705950). The contribution to disease of the risk allele in these cohorts is still under investigation [86, 87]. Interestingly, the SNP was recently shown to be associated not only with IPF, but also with NSIP in a small German cohort, which suggests that IPF and NSIP have similar pathogenesis with regard to MUC5B [87].

## 10. Airway Involvement

In the lung, MUC5B is preferentially expressed by distal airway epithelium, but not by alveolar epithelium [88]. Several exogenous factors, including cigarette smoke, and endogenous factors have been shown to increase MUC5B expression or decrease clearance in the lung [50, 89, 90]. Chronic airway diseases are commonly accompanied by raised expression of gel-forming mucins. Interestingly, in human bronchial cells it was shown that proinflammatory cytokines IL-1*β* and IL-17A were potent inducers of* MUC5B* mRNA expression. The induction by IL-1B was both time and dose dependent and involved IL-1R1 receptor binding followed by NF-*κ*B-based transcriptional mechanism [91].

The MUC5B protein is present in IPF lesions and IPF patients had significantly increased expression of* MUC5B* in the lungs compared with controls. Changes in MUC5B levels have been suggested to interfere in alveolar repair in IPF, but this needs further investigation [83, 92]. More evidence is available regarding MUC5B dependent changes in pulmonary immune regulation.* Muc5b* deficient mice have impaired mucociliary clearance. And absence of Muc5b caused accumulation of apoptotic macrophages, impaired phagocytosis, and chronic infection.* Muc5b* overexpression in mice leads to improved macrophage function [93]. In IPF patients carrying the risk allele a lower bacterial burden was found, suggesting a direct relationship between host immunity and bacterial load [94]. This beneficiary effect of the risk allele corresponds well with significant associations with improved survival in IPF [95], less severe pathological changes in FIP [92], and slower decline in FVC for IPF [96]. All evidence so far points towards a beneficiary effect of the risk allele during disease but its role in disease susceptibility remains elusive.

## 11. Genome-Wide Association Studies

The first common variant that was associated with IIP through a genome-wide association study (GWAS) was rs2736100 in the* TERT* gene [97] in a Japanese population. The association was replicated in a second GWAS including non-Hispanic white IIP patients [98] and in a Mexican candidate gene study [86]. Furthermore, three novel IIP-associated loci that were identified in the second GWAS were also replicated in the Mexican study: polymorphisms rs6793295 (*LRRC34*), rs2609255 (*FAM13A*), and rs2034650 (*IVD*) (Table 2) [86]. The* IVD* variant was also associated with IPF in a Korean IPF population [86]. For all variants, allele frequencies differed significantly between populations, but the allele associated with increased risk for IIP was consistent.

LRRC34 is of unknown function, but the LRRC34 gene is located near the* TERC* gene [98], perhaps indicating an association with telomere maintenance. Polymorphisms in the* FAM13A* gene have been associated with lung function in the general population, as well as with various lung diseases. The function of* FAM13A* is unknown, but it is speculated that* FAM13A* polymorphisms affect rho GTPases activity, possibly affecting the lung endothelial barrier [99]. The* IVD* gene encodes a mitochondrial matrix enzyme involved in leucine metabolism [98]. Why an* IVD* polymorphism is associated with IIP remains to be determined.

One further large GWAS conducted by Noth and coworkers [100] included IPF patients and controls, distributed over three stages. There might have been a partial overlay between cases from this GWAS and the previously mentioned GWAS by Fingerlin et al. [98], as a proportion of patients were recruited from the same cohorts. This study did not identify new risk alleles that have been replicated independently.

## 12. Genome Region 11p15.5

The GWAS by Noth et al. [100] identified three polymorphisms in the* TOLLIP* gene that were significantly associated with IPF. TOLLIP is a negative regulator of the TGF-beta pathway and interacts with Toll-like receptors and with interleukin-1 receptor trafficking, which makes it an interesting candidate gene for IPF susceptibility [100]. However, the association with* TOLLIP* has not been replicated independently. In the GWAS by Fingerlin et al. [98] associated* TOLLIP* SNPs had also been identified, but they discovered that the effect disappeared after correction for the effect of* MUC5B*. This suggests that there is linkage disequilibrium (LD) between* TOLLIP* and* MUC5B*. Both genes are located in the same chromosomal region, 11p15.5, just 12 kb apart (http://www.ncbi.nlm.nih.gov/gene/). Genes separated by 12 kb are considered to be in very close proximity, because the expected extension of LD in humans of European origin is at least 60 kb [101]. Measures for LD describe the nonrandom association of genetic markers based on the frequencies of the marker alleles. LD is often represented as the correlation coefficient *r* ^2^ between markers. In the study by Noth et al., *r* ^2^ between* TOLLIP* and* MUC5B* SNPs was found to be very low, *r* ^2^ < 0.16, and analysis of* TOLLIP* was therefore pursued [100]. However, another measurement for linkage disequilibrium, *D*′, provides information about the recombination breakpoints of chromosomes. SNPs with low *r* ^2^ values can reside in a linkage disequilibrium block with a high level of *D*′ between markers. In such a case disease associations are not independent [102]. Further studies are therefore needed to fully understand the contribution of the 11p15.5 region to disease.

## 13. Genes in Disease Pathogenesis

Genetic variations in surfactant associated genes* SFTPC*,* SFTPA2*, and* ABCA3* point out AEC type II dysfunction at the initiation of disease. Coping with additional epithelial damage requires proliferation of AEC type II with proper telomere maintenance controlled by* TERT*,* TERC*,* DKC1*,* TINF2*,* RTEL1*, and* PARN*. In families, one mutation that alters the quality or quantity of any of these genes is enough to cause pulmonary fibrosis. In sporadic patients, more subtle effects of a polymorphism in* TERT* might steer damage control into a similar direction, although this would likely require additional damaging environmental or genetic influences. On the other hand, it is more difficult to place the* MUC5B* association into this model of disease pathogenesis. Verified effects of the polymorphism, such as increased production that enhances the immunological properties of the lung, associate well with the observed beneficial consequences of carriership in patients, such as increased survival and slower decline of lung function. However, it does not explain the disease predisposing effect.

## 14. A Unifying Theory: MUC5B Alters Alveolar Surfactant Fluid Properties at Areas with Maximal Mechanical Stress

Interestingly, the* MUC5B* risk allele associated with the presence of interstitial lung abnormalities on HRCT in a general population. The association was independent of smoking history and stronger in a subgroup with CT evidence of fibrosis and in older participants [103]. This suggests a general role for MUC5B in induction of CT patterns of interstitial pneumonia. Such a general observation is likely to be caused by a mechanism that is uniformly present in the human lung. This mechanism might already be identified. Mechanical stress due to respiratory lung movements has been proposed to contribute to IPF and induce such CT patterns [104].

**Figure 1:**
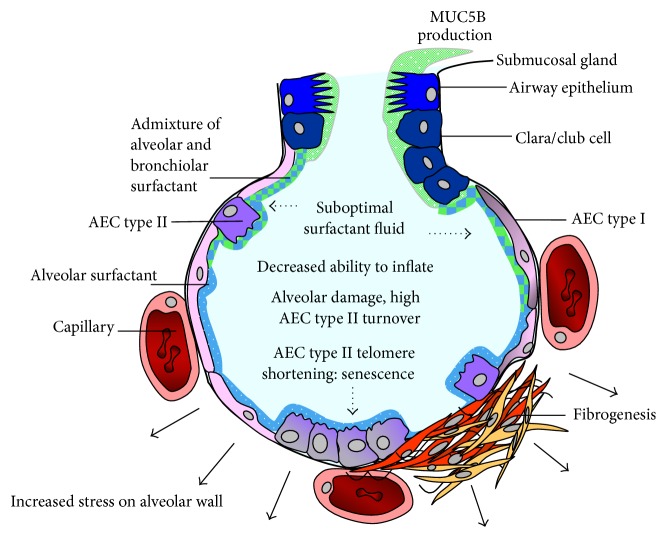
Hypothesized scheme of increased mechanical stress in* MUC5B* risk allele carriers. At areas with maximal mechanical stress in the lung, optimal surface tension lowering capacity of alveolar surfactant fluid is required. Through breathing mechanics admixture of alveolar and airway surfactant occurs. Increased amounts of MUC5B protein in* MUC5B* risk allele carriers have detrimental effect on surface tension lowering capacity of the alveolar surfactant fluid. In case of suboptimal surfactant, inflation requires increased traction on the alveolar wall and induces epithelial damage. Alveolar repair causes high epithelial cell turnover with consequent critical shortening of telomeres, which in turn induce senescence of alveolar epithelial type II cells.

Mathematical modelling showed that the distribution of IPF lesions on HRCT coincides with the hypothetical distribution of maximal mechanical stress [105]. We postulate that, in lung, where* MUC5B *is abundantly expressed, increased admixture of airway fluid to alveolar surfactant fluid might occur, thereby increasing MUC5B levels in surfactant fluid. The high MUC5B levels in the surfactant fluid might cause a significant change in its surface tension lowering capacity. Optimal surfactant reduces the surface tension by a factor of about 15 [106], which is necessary for proper alveolar size regulation during inspiration and expiration. We hypothesize that a suboptimal surfactant mix is most abundant and detrimental at areas with maximal mechanical stress as the surface tension lowering capacity of alveolar surfactant fluid is most important in these areas. Increased mechanical stress causes alveolar damage that will initially be repaired by AEC type II. Increased turnover of AEC type II is associated with decreased telomere length which in turn will lead to AEC type II senescence (Figure 1). Further experiments are necessary to confirm this hypothesis.

## Figures and Tables

**Table 1:** Mutated genes in IIP, expressing pulmonary cells, function, and mutational consequences.

Gene	Expressing cells in lung	Gene function^*∗*^	Mutated domain	Effect of mutation	Pathological process
*SFTPC*	AEC type II [107]	Component of surfactant fluid Lower surface tension [107]	Linker domain associates with LB [31]	Toxic gain of function [107]	Alteration of trafficking, dysregulation of proteostasis [31] Alteration of surfactant lipid composition [31]
			C-terminal BRICHOS domain folded in ER and GA [108]		ER stress and UPR upregulation [109–111] Activation of immune cells [112] Alteration of surfactant lipid composition [112]

*SFTPA2*	AEC type II Clara/club cell Submucosal gland [113–116]	Collectin (to modulate innate and adaptive immunity) [107, 117]	Carbohydrate-recognition domain [32]	Toxic gain of function [118]	Increase of ER stress [118] UPR upregulation [35, 119]

*ABCA3*	AEC type II [120]	Limiting membrane protein of lamellar body [107]	Intracellular loop, extracellular domain 2 [45, 46, 121]	Loss of function Toxic gain of function [107]	Abnormal surfactant processing/trafficking [45, 122] Loss of epithelial function [41] Increase of ER stress [41, 44]

*TERT*	AEC type II [123] Lung fibroblasts [124, 125] Lung epithelial cells [126]	Enzyme in telomerase complex maintaining telomere length [127–130]	All [55, 56, 60, 131–135]	Haploinsufficiency [56, 136, 137]	Telomere shortening [55, 56, 61, 62, 124, 131, 138, 139]

*TERC*	AEC type II [70, 123, 140] Lung fibroblasts [124, 125]	Template in telomerase complex [127–130]	Pseudoknot, CR4-CR5, and ScaRNA domains [55, 56, 61, 133, 139, 141, 142]	Haploinsufficiency [136, 137, 143, 144]	Telomere shortening [55, 56, 61, 124, 131, 138]

*DKC1*	Lung tissue [145]	Dyskerin (stabilizes template in telomerase complex) [64]	Near RNA-binding domain [64], near N-terminus [146–149]	X-linked loss of function [64]	Telomere shortening [64] Decrease in TERC [146]

*TINF2*	Lung tissue [150]	Telomere maintenance by shelterin complex [151]	Exon 6 residues 269–298 [63, 152, 153]	Dominant negative [63]	Telomere shortening [63]

*RTEL1*	Not detectable in lungs [154, 155]	DNA helicase, telomere T-loop, and G4 unwinding [156]	Various domains, that is, helicase and harmonin [65–67, 157]	Haploinsufficiency [66]	Telomere shortening [65–67]

*PARN*	Lung tissue [158]	Exoribonuclease controls mRNA stability [159]	Mainly CAF1 ribonuclease domain [67]	Haploinsufficiency [67]	Telomere shortening [67] Reduction of DKC1, TERT, TERC, and TERF1 [159]

^*∗*^Gene function suggested to be involved in IIP pathogenesis.

**Table 2:** Genes with polymorphisms predisposing to IIP in multiple studies, expressing pulmonary cells, function, and mutational consequences.

Gene	Expressing cells in lung	Gene function	Risk allele	Effect of risk allele	Cellular consequence
*IL1RN*	Bronchus epithelium [160] Alveolar epithelium [161] Immune cells [162]	Inhibitor of proinflammatory effect of IL-1*α* and IL-1*β* [163]	VNTR^*∗*^2 haploblock [78]^#^	Decreased expression [78]	Increase of IL-1*β*/Il-1Ra ratio with proinflammatory/fibrotic effect [74]

*TERT*	AEC type II [123] Lung fibroblasts [124] Lung epithelial cells [126]	Enzyme in telomerase complex maintaining telomere length [127–130]	rs2736100 major A allele [86, 97, 98, 164]	?	?

*MUC5B*	Airway submucosal glands [165] Macrophages [166]	Influence on rheological properties of airway mucus, mucociliary transport, and airway defense [93, 165]	rs35705950 minor T allele [83, 84, 86, 87, 96, 98, 100, 164, 167–169]	Expression ↑ [83]	Lower bacterial burden [93, 94] Improved macrophage function [93]

*LRRC34*	Not detected in human lung [170]	?	rs6793295 minor C allele [86, 98]	?	?

*FAM13A*	Lung tissue [99]	?	rs2609255 minor G allele [86, 98]	? No upregulation in lung tissue [98]	?

*IVD*	?	Mitochondrial matrix enzyme involved in leucine catabolism [98]	rs2034650 major T allele [86, 98]	? No upregulation in lung tissue [98]	?

^#^Pooled meta-analysis of five independent cohorts [74–77].
